# Community engagement in the provision of culturally competent HIV and STI prevention services: lessons from the French experience in the era of PrEP

**DOI:** 10.1002/jia2.25350

**Published:** 2019-08-30

**Authors:** Daniela Rojas Castro, Rosemary M Delabre, Stéphane Morel, David Michels, Bruno Spire

**Affiliations:** ^1^ Coalition PLUS Community‐Based Research Laboratory Pantin France; ^2^ Aix Marseille Univ INSERM, IRD, SESSTIM Sciences Economiques & Sociales de la Santé & Traitement de l'Information Médicale Marseille France; ^3^ AIDES Pantin France

**Keywords:** community, community‐based research, HIV prevention, key and vulnerable populations, PrEP, STI

Communities have been a driving force in the response to the HIV epidemic, advocating for research, the access to treatment and healthcare, and human rights for key populations (KP) and people living with HIV (PLHIV). The importance of community engagement (CE) in the development and implementation of pertinent programmes throughout the HIV care continuum has been widely recognized 1, 2, 3. In the context of increasing pre‐exposure prophylaxis (PrEP) research, interest and access (though still limited), there is an opportunity to have a fresh look at CE regarding HIV/STI research and care delivery. France, where PrEP has been authorized and fully reimbursed since 2016, may provide key lessons for CE in the provision of comprehensive, culturally adapted HIV/STI prevention and treatment services.

Community involvement in HIV/AIDS is political and ethical. Community‐based organizations (CBOs) such as Gay Men's Health Crisis (US), Terrence Higgins Trust (UK), the Grupo Pela Vidda (Brazil), AIDES (France), or international organizations such as ACT‐UP, have historically played important roles in advocating for suitable information on prevention tools and adequate access to health for PLHIV and most‐at‐risk populations 2, 3, 4. PrEP research is not an exemption 5. For example, Act Up‐Paris and others advocated for the early termination of two PrEP studies due to, among other reasons, the lack of medical services for those who seroconverted on study 6, 7, 8, 9. While implementation of “Good Participatory Practice Guidelines” 10, 11 and community advisory boards 12 in research studies are steps forward, further effort is needed to ensure more meaningful CE throughout the entire life course of research studies 13, 14. For example, by building the evidence‐base for CE and evaluating its success in meeting community needs 15.

In 2008, AIDES adopted a unique strategy to invest financial and human resources for the creation of a community‐based research unit. Working in partnership with research institutions and funding bodies, community‐based studies have identified community needs and contributed to the development of innovative and adapted services: rapid HIV testing, educational sessions for injection drug users, and PrEP counselling.

While medical providers may lack the time, skill and/or motivation to address sexual health issues 16, 17, CBOs are well‐placed to identify the sexual health needs of KP and provide comprehensive and adapted care 18. The Fenway Community Health Center in Boston provides comprehensive “culturally competent” care 19. The 56 Dean Street clinic in London offers a succesful well‐being programme and an “express” service for self‐sampling HIV and STI tests 20. In Bamako, the CBO ARCAD‐SIDA's night sexual health clinic provides testing and treatment services for MSM and sex workers 21. Finally, results of a community‐based testing satisfaction survey conducted by AIDES 22 partially led to the creation of two community‐based sexual health structures that integrate sexual and mental health consultations (SPOT Beaumarchais in Paris and SPOT Longchamp in Marseille). Community‐based clinical programmes are important examples of how communities and medical professionals may work together to develop and provide effective services.

PrEP provision is an opportunity to provide comprehensive sexual health services, engage individuals on their needs, and to equip them to better evaluate and reduce their HIV/STI risk. AIDES has been a full partner in two PrEP studies: ANRS‐Ipergay 23 and ANRS‐Prévenir 24, 25. Peer counselling, provided by AIDES counsellors, was constructed collectively with social science researchers (GRePS and Inserm). Based upon individual needs and expectations, discussions go beyond purely medical aspects regarding PrEP to include questions such as “What risks can you identify related to your sex life?” and “Is your sex life as fulfilling as you would like?.” Therefore, PrEP is not an end in itself, but rather an opportunity to empower communities regarding sexual health.

As PrEP protects from HIV but not STIs, appropriate and adapted risk reduction methods such as prophylactic antibiotics 26 should be considered. Follow‐up appointments, required in the provision of PrEP, allow for STI information, regular screenings and early treatment. However, this regular hospital medical follow‐up can represent a barrier, and respondents to a European community‐based survey felt that PrEP should be available at community‐based health settings or at the general practitioners’ 27. Provision of HIV and STI services outside of traditional medical structures is essential to reach populations who are most exposed and face access barriers. Community‐based initiatives such as community‐based testing have reached at‐risk populations as well as those who have never been tested 18, 28 and have identified individuals at an earlier disease stage 29. More innovative partner notification strategies, such as CheckOut™ developed by the Checkpoint LX in Portugal 30, may be used in the context of PrEP 25.

All communities particularly affected by HIV and STIs must be involved in the development of adapted and inclusive information and programmes regarding provision of PrEP and/or other services (e.g. PEP, STI prophylaxis) which reach KP other than MSM. Regarding transgender people, for example, concerns related to finding “trans‐competent” providers and potential interaction with hormones should be addressed 31. The Thai Red Cross Tangerine Health Center is one example of a community‐engaged model providing comprehensive services for transgender women 32. Women may experience barriers to PrEP, indicating a need for adapted services. Several community‐based initiatives are increasingly providing tailored PrEP information to increase awareness among women 33, 34. CE is also critical for the development of adapted and sustainable prevention programmes among sex workers 35, 36. Finally, it is necessary to address stigma related to sexual preferences, drug use, sex work and PrEP use 37, 38, 39.

Communities have the knowledge, skills and motivation to provide culturally adapted information and services for PLHIV and KP. Community‐based initiatives can and must go further. For example, community‐based ART delivery, already implemented in some southern countries 40, needs to be expanded to northern countries. Partnerships between communities and traditional health structures will require the support of governments and international bodies to implement and enforce policies for task shifting in addition to significant funding. We call for a united effort amongst government bodies, health providers, and CBOs to make a comprehensive, positive approach to sexual health for PLHIV and for those most exposed to HIV a reality.

## Competing interests

The authors declare that they have no competing interests.

## Authors’ contributions

BS, DRC, RMD, SM and DM conceptualized the commentary. SM and DM provided content on AIDES’ community‐based approach and activities. BS, DRC, RMD, SM and DM discussed key ideas and concepts forming the basis of this commentary. RMD and DRC reviewed the literature and wrote the manuscript. All authors reviewed and approved the final version.

## References

[1] UNAIDS . The essential role of civil society. Report on the Global AIDS Epidemic. A UNAIDS 10th Anniversary Special Edition. Geneva; 2006. 202–22.

[2] Trapence G , Collins C , Avrett S , Carr R , Sanchez H , Ayala G , et al. From personal survival to public health: community leadership by men who have sex with men in the response to HIV. Lancet. 2012;380(9839):400–10. 22819662 10.1016/S0140-6736(12)60834-4 PMC3805044

[3] Merson MH , O'Malley J , Serwadda D , Apisuk C . The history and challenge of HIV prevention. Lancet. 2008;372(9637):475–88. 18687461 10.1016/S0140-6736(08)60884-3

[4] Aggleton P , Pedrosa JS . Community, solidarity and action—grupo pela VIDDA, Brazil. AIDS Care. 1994;6(3):343–8. 7948090 10.1080/09540129408258646

[5] Sugarman J , Mayer KH . Ethics and pre‐exposure prophylaxis for HIV‐infection. J Acquir Immune Defic Syndr. 2013;63:S135–9. 23764625 10.1097/QAI.0b013e3182987787 PMC3728665

[6] Ahmad K . Trial of antiretroviral for HIV prevention on hold. Lancet Infect Dis. 2004;4(10):597. 15470760 10.1016/s1473-3099(04)01159-4

[7] Forbes A , Mudaliar S . Preventing Prevention trial failures: a case study and lessons for future trials from the 2004 tenofovir trail in Cambodia [Internet]. Global Campaign for Microbicides; 2009 [cited 2018 Dec 19]. Available from: https://path.org/resources/preventing-prevention-trial-failures-a-case-study-and-lessons-for-future-trials-from-the-2004-tenofovir-trail-in-cambodia/

[8] Singh JA , Mills EJ . The abandoned trials of pre‐exposure prophylaxis for HIV: what went wrong? PLoS Med. 2005;2(9):e234. 16008507 10.1371/journal.pmed.0020234 PMC1176237

[9] McGrory E , Irvin A , Heise L . Research rashomon: lessons from the Cameroon pre‐exposure prophylaxis trial site [Internet]. Global Campaign for Microbicides; 2009 [cited 2018 Dec 19]. Available from: https://www.path.org/resources/research-rashomon-lessons-from-the-cameroon-pre-exposure-prophylaxis-trial-site/

[10] UNAIDS, AVAC . Good participatory practice guidelines for biomedical HIV prevention trials. 2007.

[11] UNAIDS, AVAC . Good Participatory Practice (GPP) Guidelines. 2011.

[12] Morin SF , Morfit S , Maiorana A , Aramrattana A , Goicochea P , Mutsambi JM , et al. Building community partnerships: case studies of Community Advisory Boards at research sites in Peru, Zimbabwe, and Thailand. Clin Trials Lond Engl. 2008;5(2):147–56. 10.1177/1740774508090211 18375653

[13] Day S , Blumberg M , Vu T , Zhao Y , Rennie S , Tucker JD . Stakeholder engagement to inform HIV clinical trials: a systematic review of the evidence. J Int AIDS Soc. 2018;21(S7):e25174. 30334358 10.1002/jia2.25174 PMC6192899

[14] Fregonese F . Community involvement in biomedical research conducted in the global health context; what can be done to make it really matter? BMC Med Ethics [Internet]. 2018 Jun 15 [cited 2019 May 13];19 Suppl 1 Available from: https://www.ncbi.nlm.nih.gov/pmc/articles/PMC6019999/ 10.1186/s12910-018-0283-4 PMC6019999 29945597

[15] MacQueen KM , Bhan A , Frohlich J , Holzer J , Sugarman J ; Ethics Working Group of the HIV Prevention Trials Network . Evaluating community engagement in global health research: the need for metrics. BMC Med Ethics. 2015;16:44. 26126899 10.1186/s12910-015-0033-9 PMC4488111

[16] Carter JW , Hart‐Cooper GD , Butler MO , Workowski KA , Hoover KW . Provider barriers prevent recommended sexually transmitted disease screening of HIV‐infected men who have sex with men. Sex Transm Dis. 2014;41(2):137–42. 24413496 10.1097/OLQ.0000000000000067

[17] Hoover KW , Butler M , Workowski K , Carpio F , Follansbee S , Gratzer B , et al. STD screening of HIV‐infected MSM in HIV clinics. Sex Transm Dis. 2010;37(12):771–6. 20585275 10.1097/OLQ.0b013e3181e50058

[18] Champenois K , Le Gall J‐M , Jacquemin C , Jean S , Martin C , Rios L , et al. ANRS–COM'TEST: description of a community‐based HIV testing intervention in non‐medical settings for men who have sex with men. BMJ Open [Internet]. 2012 [cited 2018 Dec 21];2(2). Available from: https://www.ncbi.nlm.nih.gov/pmc/articles/PMC3323802/ 10.1136/bmjopen-2011-000693 PMC3323802 22466158

[19] Mayer K , Appelbaum J , Rogers T , Lo W , Bradford J , Boswell S . The evolution of the Fenway Community Health model. Am J Public Health. 2001;91(6):892–4. 11392929 10.2105/ajph.91.6.892 PMC1446463

[20] Kirby T , Thornber‐Dunwell M . Dean Street clinics‐battling London's MSM HIV epidemic. Lancet HIV. 2018;5(5):e210. 29739698 10.1016/S2352-3018(18)30070-5

[21] Coulibaly A , Dembelé Keita B , Henry E , Trenado E . Faciliter l'accès aux soins des populations les plus exposées: l'expérience de la clinique nocturne de santé sexuelle de Bamako au Mali. Promot Santé En Afr. 2014;Juillet:67–70. 25380379

[22] Rios L , Rojas Castro D , Quatremère G , Monvoisin D , Fugon L , Tessier S , et al. Enquête d'opinion sur la qualité d'offre de dépistage communautaire de l'association AIDES et autres attentes de services de santé sexuelle. Poster presentation at: AFRAVIH; 2014; Montpellier, France.

[23] Molina J‐M , Capitant C , Spire B , Pialoux G , Cotte L , Charreau I , et al. On‐demand preexposure prophylaxis in men at high risk for HIV‐1 infection. N Engl J Med. 2015;373(23):2237–46. 26624850 10.1056/NEJMoa1506273

[24] ANRS . L’étude ANRS Prévenir démarre [Internet]. 2017 [cited 2019 Jan 7]. Available from: http://www.anrs.fr/fr/actualites/313/letude-anrs-prevenir-demarre

[25] Morel S . Promoting sexual health through community education and activism: How community involvement could improve sexual health access & STI test regularity. Presentation at: STI 2018: Understanding and Addressing the HIV and STI Syndemics; 2018; Amsterdam, The Netherlands.

[26] Molina J‐M , Charreau I , Chidiac C , Pialoux G , Cua E , Delaugerre C , et al. Post‐exposure prophylaxis with doxycycline to prevent sexually transmitted infections in men who have sex with men: an open‐label randomised substudy of the ANRS IPERGAY trial. Lancet Infect Dis. 2018;18(3):308–17. 29229440 10.1016/S1473-3099(17)30725-9

[27] Bernier A , Delabre R , Schlegel V , Vilotitch A , Ghosn J . What role might general practitioners play in pre‐exposure prophylaxis programs in Europe? Results from a 2016 European community‐based survey “Flash PrEP in Europe” (FPIE). Poster presented at: 16th European AIDS Conference; 2017; Milan, Italy.

[28] Fernàndez‐López L , Reyes‐Urueña J , Agustí C , Kustec T , Klavs I , Casabona C . The COBATEST network: a platform to perform monitoring and evaluation of HIV community‐based testing practices in Europe and conduct operational research. AIDS Care. 2016;28(sup1):32–6. 10.1080/09540121.2016.1146218 PMC4828621 26883807

[29] Suthar AB , Ford N , Bachanas PJ , Wong VJ , Rajan JS , Saltzman AK , et al. Towards universal voluntary HIV testing and counselling: a systematic review and meta‐analysis of community‐based approaches. PLoS Med. 2013;10(8):e1001496. 23966838 10.1371/journal.pmed.1001496 PMC3742447

[30] Rocha M , Guerreiro R , Pinto N , Rojas J , Ferreira F , Esteves J , et al. Digital Partner Notification Service at a Community‐based Voluntary Counselling and Testing Centre for Men Who Have Sex with Men: Checkpoint LX, Lisbon, Portugal. Poster presentation at: 21st International AIDS Conference (AIDS2016); 2016; Durban.

[31] Sevelius JM , Keatley J , Calma N , Arnold E . ‘I am not a man’: trans‐specific barriers and facilitators to PrEP acceptability among transgender women. Glob Public Health. 2016;11(7–8):1060–75. 26963756 10.1080/17441692.2016.1154085 PMC10204128

[32] Janamnuaysook R . The Importance of Trans Comprehensive Health Services to the HIV Responses: Lessons Learned from TANGERINE Community Health Center [Internet]. 2019Apr 24 [cited 2019 May 13]. Available from: http://www.weareaptn.org/2019/04/24/the-importance-of-trans-comprehensive-health-services-to-the-hiv-responses-lessons-learned-from-tangerine-community-health-center/

[33] Women and PrEP . Women and PrEP [Internet]. 2019 [cited 2019 Jun 22]. Available from: http://womenandprep.org.uk/

[34] Boerner H . To Halt HIV, Advocates Push For PrEP Outreach To Black Women [Internet]. NPR.org. 2019 [cited 2019 Jun 22]. Available from: https://www.npr.org/sections/health-shots/2019/02/08/691740052/to-halt-hiv-advocates-push-for-prep-outreach-to-black-women

[35] Bekker L‐G , Johnson L , Cowan F , Overs C , Besada D , Hillier S , et al. Combination HIV prevention for female sex workers: what is the evidence? Lancet. 2015;385(9962):72–87. 25059942 10.1016/S0140-6736(14)60974-0 PMC10318470

[36] Shannon K , Crago A‐L , Baral SD , Bekker L‐G , Kerrigan D , Decker MR , et al. The global response and unmet actions for HIV and sex workers. Lancet Lond Engl. 2018;392(10148):698–710. 10.1016/S0140-6736(18)31439-9 PMC6384122 30037733

[37] Spire B , de Zoysa I , Himmich H . HIV prevention: what have we learned from community experiences in concentrated epidemics? J Int AIDS Soc. 2008;11:5. 19014656 10.1186/1758-2652-11-5 PMC2584058

[38] Pinto RM , Berringer KR , Melendez R , Mmeje O . Improving PrEP implementation through multilevel interventions: a synthesis of the literature. AIDS Behav. 2018;22(11):3681–91. 29872999 10.1007/s10461-018-2184-4 PMC6208917

[39] Sawicki DA , Meffert BN , Read K , Heinz AJ . Culturally competent health care for sex workers: an examination of myths that stigmatize sex‐work and hinder access to care. Sex Relatsh Ther [Internet]. 2019 Feb 19 [cited 2019 Jun 23]. Available from: https://www.ncbi.nlm.nih.gov/pmc/articles/PMC6424363/ 10.1080/14681994.2019.1574970 PMC6424363 30899197

[40] Decroo T , Rasschaert F , Telfer B , Remartinez D , Laga M , Ford N . Community‐based antiretroviral therapy programs can overcome barriers to retention of patients and decongest health services in sub‐Saharan Africa: a systematic review. Int Health. 2013;5(3):169–79. 24030268 10.1093/inthealth/iht016

